# Immunomodulatory effects of atorvastatin on MRL/lpr mice

**DOI:** 10.1186/s42358-022-00282-z

**Published:** 2022-12-05

**Authors:** Jing Sun, Weidong Xu, Zhiying Wu, Caijin Cao, Yane Tan, Meifang Zhu, Hongze Wu, Jianping Yu

**Affiliations:** 1 Clinical Medical College, Jiangxi University of Chinese Medicine, Nanchang, 330001 China; 2 Department of Rheumatology, Jiujiang Hospital of Chinese Medicine, Jiujiang, 332099 China; 3 Department of Rheumatology, The Affiliated Hospital of Jiangxi University of Chinese Medicine, Nanchang, 330001 China

**Keywords:** Atorvastatin, Systemic lupus erythematosus, B cell, Immunomodulatory effects, MRL/lpr mice

## Abstract

**Background:**

Statins have long been extensively prescribed as effective lipid-lowering agents, but statins have also been recognized as novel immunomodulators in recent years. This study was designed to investigate the immunomodulatory effects of atorvastatin on lupus-prone MRL/lpr mice.

**Methods:**

A total of 30 8-week-old female MRL/lpr mice were randomly divided into three groups and orally administered vehicle, atorvastatin orhydroxychloroquine sulfate for 11 weeks. In vivo, the effects of atorvastatin on the survival rate, renal function and spleen index in MRL/lpr mice were examined. Ex vivo, splenic B-cell proliferation was assessed by a Cell Counting Kit-8.

**Results:**

Oral atorvastatin failed to prolong survival time, or reduce the levels of proteinuria, or serum anti-dsDNA antibody and complement proteins (C3, C4). Histologically, no significant improvement by atorvastatin was observed in the pathological manifestations of renal damage, while hydroxychloroquine sulfate significantly improved glomerular injury. Ex vivo, atorvastatin suppressed the proliferation of splenic B lymphocytes.

**Conclusion:**

Oral atorvastatin monotherapy had no therapeutic effects on MRL/lpr mice, whereas atorvastatin inhibited splenic B-cell proliferation in vitro, suggesting that atorvastatin has a potential therapeutic effect on systemic lupus erythematosus.

## Introduction

Systemic lupus erythematosus (SLE) is a chronic autoimmune disease characterized by the production of multiple autoantibodies, which can affect multiple organs and present with a variety of clinical manifestations. SLE is more common among African American women and other minority women [1, 2]. Currently, patients with SLE are treated with glucocorticoids, nonsteroidal anti-inflammatory drugs, antimalarial agents, and immunosuppressive drugs, including cyclophosphamide, methotrexate, hydroxychloroquine, and mycophenolate mofetil [2, 3]. These drugs have achieved varying potency, but also produced numerous side effects.

Statins, or inhibitors of HMG-CoA reductase, have been widely used for lowering total cholesterol, LDL-cholesterol and triglycerides [4–6]. The pleiotropic effect of statins has been recognized for years [7]. Its role in the prevention of cardiovascular disease for at least 3–4 decades. Fasano recently ratified that statins are effective in preventing cardiovascular diseases [8]. Several studies have shown that statins have immunomodulatory effects on inflammation and autoimmune diseases, including multiple sclerosis, rheumatoid arthritis, and graft-versus-host disease [9–11]. In addition, Watanabe et al. described the protective thrombotic effect of a statin for SLE and antiphospholipid syndrome, confirming the value of this drug as an adjunct in the treatment of autoimmune diseases [12]. The immunomodulatory action of statins in autoimmune diseases, despite being the subject of several studies, has not been effectively proven, given the great phenotypic variability of these diseases and their complex pathophysiology. Specifically, with regard to systemic lupus erythematosus, a recently published meta-analysis did not prove that statins would be able to promote the control of this serious disease [13]. An initial report demonstrated that statins inhibit the IFN-γ-induced expression of MHC-II and subsequent T-cell activation [14]. In 2004, Lawman demonstrated that autoreactive B cells and the development of lupus disease in NZB/W mice were inhibited by intraperitoneal injection of atorvastatin for 14 consecutive days [15]. Four years later, Graham discovered no protective effect in NZB/W mice, as oral atorvastatin was administered daily for 20 weeks [16]. However, to our knowledge, there are no data showing a possible immunomodulatory effect of atorvastatin on MRL/lpr mice, an accepted model of human SLE or lupus, which spontaneously develop an autoimmune disorder characterized by immune complex glomerulonephritis, vasculitis, arthritis, and autoantibodies [17, 18].

In this study, we used an MRL/lpr mouse model of spontaneous SLE to dissect the role of atorvastatin in survival time and to explore its therapeutic potential for SLE.

## Materials and methods

### Mice

4-week-old female MRL/lpr mice were purchased from the SLRC Laboratory (Shanghai, China) and maintained under a specific pathogen-free facility at Jiangxi University of Chinese Medicine, with a controlled temperature (26 ℃) and on a 12-h light–dark cycle. Experiments were conducted in accordance with the National Institutes of Health Guide for Care and Use of Laboratory Animals and were approved by the Animal Care Committee of Jiangxi University of Chinese Medicine.

### Reagents

Atorvastatin and hydroxychloroquine sulfate (HCQ) were purchased from Sigma-Aldrich Co. LLC. (Shanghai, China) and SPH Zhongxi Pharmaceutical Co.,Ltd. (Shanghai, China), respectively. Atorvastatin was brought into suspension in normal saline (NS) at 0.286 mg/ml, and a 0.3-ml volume (equivalent to 2.6 mg/kg) was administered via oral gavage, once a day for six consecutive days a week. NS was orally administered as a vehicle control. The positive control group was orally administered HCQ (5 mg/kg/day). All mice (n = 30) were orally administered at 8 weeks of age for 6 consecutive days a week, and the surviving mice were killed at 19 weeks of age. The dose of atorvastatin (2.6 mg/kg/day) is about approximately the dose given to human patients [19, 20].

### Enzyme-linked immunosorbent assay (ELISA)

At the end of the experiment, blood was harvested from surviving mice by removing their eyeballs. Serum was separated from blood by centrifugation at 3500 rpm for 10 min. The levels of anti-dsDNA antibody, complement C3, and complement C4 in mouse serum were evaluated using mouse anti-dsDNA antibody, complement C3, and complement C4 ELISA kits (Shanghai Yaji Biotechnology Co., Ltd) according to the manufacturer's instructions.

### Evaluation of renal injury

The day before the mice were sacrificed, the mice were single-housed in metabolic cages for a period of 12 h with no food. Urine samples from individual mice were collected. Urinary protein contents, blood urea nitrogen (BUN) and serum albumin (ALB) levels were detected using a HITACHI-7080 automatic biochemical analyzer (Hitachi High Technologies Corporation, Tokyo, Japan).

The left kidneys were fixed in 10% formaldehyde for 24 h and embedded in paraffin. Two-micrometer sections were stained with hematoxylin and eosin. Kidney sections were scored according to reported criteria [21], which were analyzed blindly by two experienced renal pathologists.

### CCK8 assay

Cell viability was assayed using a Cell Counting Kit-8 (Yeasen Biotech Co., Ltd, Shanghai, China). At study termination, the spleens of surviving vehicle-treated control mice were harvested and weighed, and a single-cell suspension of splenocytes was prepared. After 6 h of culture, suspended spleen cells were seeded into 2 ml tubes at 10^7^ per tube, and splenic B cells were purified by negative isolation. Dynabeads mouse pan T (Thy 1.2) Kits (Invitrogen) were added to a tube at a concentration of 25 µg/ml for 30 min of incubation to bind T cells, and then the supernatant was collected after placing the tube in a magnet for 2 min to obtain B cells. After 24 h of culture, 100 µl splenic B-cell suspension respectively treaded with 3 reagents (NS, HCQ and atorvastatin) was added to a 96-well plate at a cell density of 2,500 cells per well. At the indicated time points (12 h,24 h,48 h and 72 h), 10 μl of CCK8 reagent was added to each well in four independent experiments. After 2 h of incubation, the optical densities at 450 nm were assayed by a microplate reader.

### Statistical analysis

Statistical analyses were performed using GraphPad Prism 8.0 software. Data are presented as the mean ± SEM. The log-rank test was conducted for the analysis of survival curves. One-way analysis of variance with Dunnett’s post-test was employed to assess significant differences among multiple groups. Two-way analysis of variance was employed for comparison of three groups of splenic primary B-cell viability.

## Results

### Effects of atorvastatin on survival rate, renal function, and spleen index in MRL/lpr mice

**Fig. 1:**
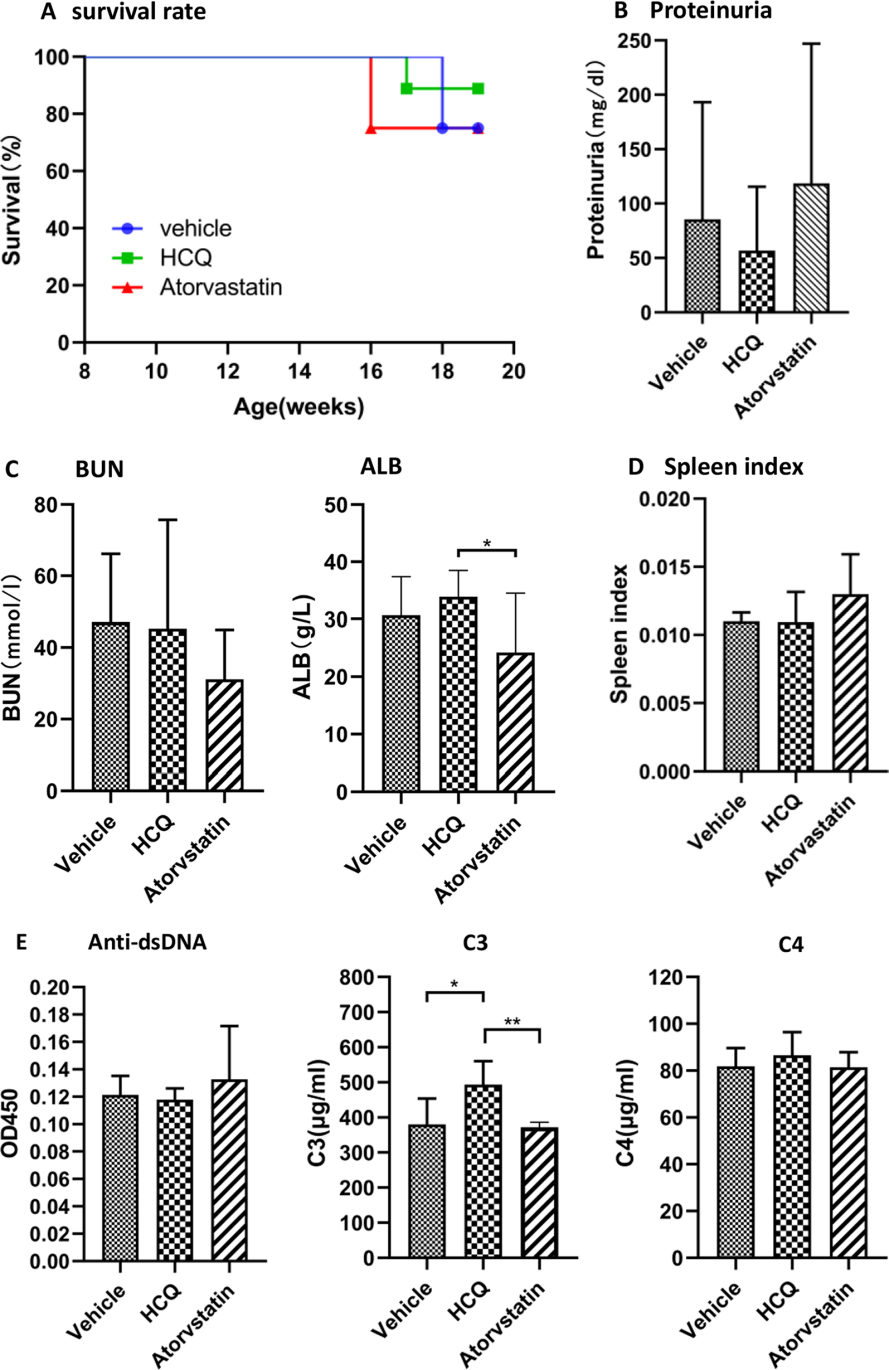
Survival, proteinuria, blood urea nitrogen (BUN), albumin (ALB) and spleen index in female MRL/lpr mice treated with atorvastatin and HCQ. Eight-week-old female MRL/lpr mice were orally administered with normal saline (vehicle), HCQ (positive control), or atorvastatin, once daily, 6 consecutive days a week for 11 weeks. **A** Cumulative survival rate of female MRL/lpr mice for 11 weeks. **B** The proteinuria level was measured at the end of the experiment. **C** The serum levels of BUN and ALB were assayed at the end of experiment. **D** Ratios of spleen weight to body weight (spleen index). **E** The levels of IgG anti-dsDNA, C3 and C4 in the serum which were obtained from MRL/lpr mice after 11 weeks treatment, were detected by ELISA. Bars in **B**–**E** demonstrate the mean ± SEM. *P < 0.05; **P < 0.01

MRL/lpr mice are crossbred from multiple generations of closely related mice that spontaneously develop syndromes similar to human SLE, including the production of antinuclear antibody and immune complex-mediated kidney damage. To evaluate the long-term efficacy of the atorvastatin regimen, treatment of the mice lasted for 11 weeks. For the study in the MRL/lpr mouse model of SLE, an oral dose of 2.6 mg/kg/day was chosen, a dose that is moderate-intensity statin therapy for humans with hypercholesterolemia [20]. We monitored the survival of mice in each group. As shown in Fig. 1A, we observed significantly reduced mortality in HCQ-treated mice, with 88.89% survival at 19 weeks, compared with 75.00% survival in both atorvastatin-treated mice and vehicle-treated control mice. Despite the greater survival of the HCQ-treated group, the difference between groups was not statistically significant. Atorvastatin accelerated kidney damage, as presented by the increase in proteinuria levels and significant decrease in serum ALB levels (Fig. 1B, C). HCQ-treated mice, in contrast, remained relatively low level of proteinuria. At the end of the treatment, BUN increased in all groups, with a slightly lower level in the atorvastatin group (Fig. 1C). Compared with the vehicle group and HCQ group, the ratio of spleen weight to body weight was increased in mice treated with atorvastatin (Fig. 1D). However, these data were not statistically significant.

### Anti-dsDNA antibodies and complement levels

SLE is characterized by the production of large amounts of antinuclear antibodies, anti-dsDNA antibodies, and circulating immune complexes in the serum leading to a series of organ damages. Anti-dsDNA antibodies play an important pathogenic role in SLE, whereas complements play a crucial role in the dissolution of target cells, clearance of circulating immune complexes and other immune defense mechanisms [22–24]. As shown in Fig. 1E, after 11 weeks treatment, atorvastatin increased anti-dsDNA antibodies in serum derived from atorvastatin-treated MRL/lpr mice, while HCQ slightly decreased them. Serum levels of C3 and C4 were not significantly affected by atorvastatin treatment. However, oral HCQ induced a significant increase in the serum level of C3. The results demonstrate that atorvastatin did not inhibit the production of anti-dsDNA antibodies and had no impact on serum levels of C3 and C4 in SLE model mice.

### Effects of atorvastatin on renal damage

**Fig. 2:**
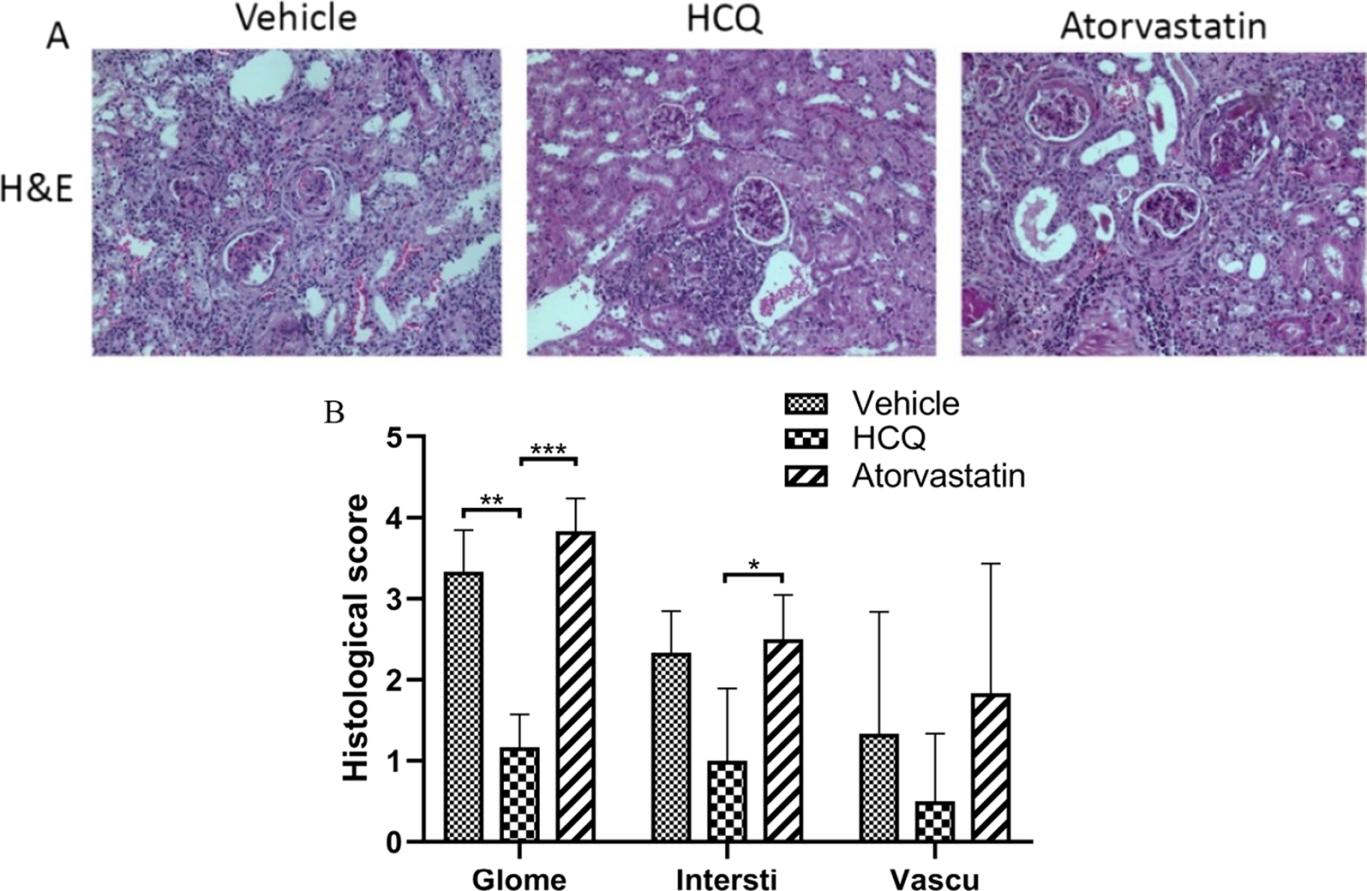
No apparent ameliorating effect of atorvastatin on renal damage. **A** Representative kidney sections stained with H&E (original magnification, × 200). **B** Histological scores for glomerulonephritis (Glome), interstitial nephritis (Intersti) and vascular (Vascu) lesions, based on H&E staining. Data are the mean ± SEM. *P < 0.05, HCQ versus Atorvastatin; **P < 0.01, vehicle versus HCQ; ***P < 0.001, HCQ versus atorvastatin

Kidney histopathology examination demonstrated that vehicle-treated mice exhibited severe renal damage, characterized by mesangial cell proliferation, glomerulosclerosis, crescent formation, tubule atrophy, tubule protein deposition, diffuse interstitial and perivascular mononuclear cell infiltration and vasculitis. The pathological lesions of the kidney in atorvastatin-treated mice were more severe in glomerular and perivascular infiltration than those in vehicle-treated mice. However, such lesions were not statistically significant. In contrast, HCQ-treated mice showed less severe renal injury with significantly decreased glomerular, interstitial, and perivascular lesions. These observations implied that atorvastatin had no apparent ameliorating effects against the development of spontaneous SLE-like kidney lesions (Fig. 2).

### Atorvastatin inhibits B cells proliferation

**Fig. 3:**
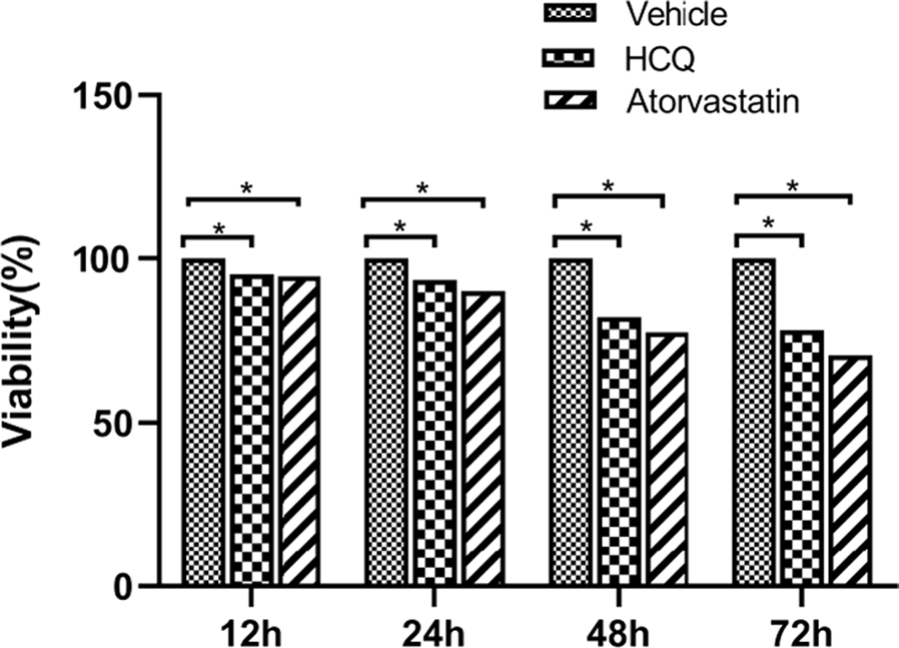
Atorvastatin inhibited B cells Proliferation. The bars show the mean values with SEM. *P < 0.05, versus vehicle

B lymphocytes play an important role in the occurrence and development of SLE. To clarify the role of atorvastatin in regulating B cells in lupus-prone mice, we performed CCK8 assay. Splenic B cells were exposed to NS, HCQ, and atorvastatin respectively, and cell viability was evaluated by CCK8 assay after treatment for 12 h, 24 h, 48 h and 72 h. As shown in Fig. 3, compared with the vehicle group, the atorvastatin group and HCQ group had lower viability of splenic B cells, and we found that the viability of the atorvastatin group showed a trend of gradual decline over time. The results indicated that atorvastatin significantly inhibited B-cell proliferation in vitro in MRL/lpr mice with spontaneous SLE.

## Discussion

At present, accumulating studies recognize atorvastatin as a new class of immunomodulators. In this study, we explored the immunoregulatory effects at oral atorvastatin of moderate-intensity doses. The results demonstrated that while atorvastatin treatment in MRL/lpr mice had no significant beneficial impact on survival, splenomegaly, serum anti-dsDNA antibody, and complement levels, or extent of renal function, it significantly inhibited in vitro B-cell proliferation.

A previous study showed that autoreactive B cells and the development of lupus disease in NZB/W mice were inhibited by atorvastatin intraperitoneally injected at a dose of 30 mg/kg for 14 consecutive days [25]. Moreover, no protective effect was observed in NZB/W mice after oral administration of atorvastatin for 20 weeks [16]. Both oral and intraperitoneal injections have been used in NZB/W mice for these SLE studies. In our study, we selected MRL/lpr mice, which spontaneously develop into a disease resembling to SLE in humans and offered a valuable opportunity to study SLE in the preclinical phase. The reason for the experiment on MRL/lpr mice lies in the hope of discovering one or more similar immunomodulatory effects that might clarify the therapeutic potential of atorvastatin in murine and ultimately human SLE. For the study in the MRL/lpr mouse model of SLE, we used an oral dose of 2.6 mg/kg/day, a dose that is moderate-intensity statin therapy for humans with hypercholesterolemia, which is different from the atorvastatin oral dose above the maximum tolerated dose used by Graham. Moderate-intensity statin therapy is widely applied for atherosclerosis prevention in SLE. At the same time, we used NS and HCQ as the blank control group and positive control group, respectively, to compare with the atorvastatin group. In MRL/lpr mice, survival was the same in the atorvastatin group as in the vehicle group, but not longer than in the HCQ group. Atorvastatin accelerated kidney damage, as presented by the increase in proteinuria levels and significant decrease in serum ALB levels. HCQ-treated mice, in contrast, retained relatively low levels of proteinuria and slightly higher levels of ALB. Renal histopathological examination further confirmed that atorvastatin-treated mice exhibited more severe glomerular and perivascular infiltration than vehicle-treated mice. In comparison, HCQ-treated mice showed less severe renal injury with significantly decreased glomerular, interstitial, and perivascular lesions. In terms of clinical symptom relief, our findings were consistent with those of NZB/W mice treated with atorvastatin orally, i.e., no therapeutic response in a different murine model of SLE. These findings are supported by a recent meta-analysis that showed no significant effect of statin treatment on the Systemic Lupus Erythematosus Disease Activity Index, but showed a reduction on plasma C-reactive protein concentration with lipophilic statins [26].

Although atorvastatin failed to show protective effects on clinical disease in our study, we conducted several assays to clarify the immunomodulatory activity of atorvastatin in MRL/lpr mice. Atorvastatin had no positive effect on serum levels of anti-dsDNA antibodies, complement C3, or C4, while HCQ induced a slight decrease in anti-dsDNA antibodies and a significant increase in the serum level of C3. We also analyzed B-cell proliferation in vitro. Atorvastatin had a significant inhibitory effect on the proliferation of B cells derived from MRL/lpr mice, as shown by in vitro experiments, which was different from atorvastatin's lackluster performance in immunoregulation in vivo. In contrast, HCQ indicated a protective effect both in vitro and in vivo. Complement activation is considered one of the pathogenesis of SLE and is often clinically manifested as decreased plasma C3 and C4 levels during lupus disease activity [24, 27, 28]. Coupled with the fact that Lawman et al. used a very high dose of atorvastatin injected intraperitoneally, 30 mg/kg, it is not known whether the megadose or the route of administration was responsible for the beneficial effect of preventing nephritis. The oral route is preferentially used in humans, and it is unlikely that doses higher than those used for the treatment of hypercholesterolemia will be tested as an immunomodulator in SLE in view of the potential for negative side effects. Whether in animal models of SLE or in human studies, usual doses of statins have not demonstrated a positive effect on disease activity, despite immunomodulatory effects being observed in vitro or in vivo. In addition, the possibility of serious adverse events when using the same dose that is used in vitro to in vivo can be as harmful and even fatal as rhabdomyolysis.

Several clinical trials have investigated the efficacy of atorvastatin in treating a variety of inflammatory diseases. Lee and colleagues reported that atorvastatin treatment significantly decreased COVID-19-related mortality [29]. Coronavirus disease-2019 (COVID-19) is thought to be a trigger of autoimmunity, as numerous recordsimply that hyperstimulation of the immune system by severe acute respiratory syndrome coronavirus 2 (SARS-CoV-2) leads to multiple types of autoantibodies [30]. A prospective study demonstrated atorvastatin reduced proteinuria and renal disease progression in idiopathic chronic glomerulonephritis patients with proteinuria, chronic kidney disease, and hypercholesterolemia [31]. The beneficial effects of statin therapy have been revealed in the treatment of inflammatory rheumatic diseases. A randomized trial enrolled 116 patients with rheumatoid arthritis and demonstrated that atorvastatin mediated anti-inflammatory effects clinically [32]. No randomized studies of patients with SLE have been reported, although case studies on 3 SLE patients reported rapidly reduced proteinuria after statin therapy [33]. Statin therapy has great potential in immune regulation and needs to be exploited.

## Conclusion

In conclusion, our study confirmed that oral atorvastatin failed to ameliorate lups-like disease in MRL/lpr mice, while atorvastatin significantly inhibited splenic B-cell proliferation in vitro.

## Data Availability

All data generated in this study are available from corresponding authors on valid request.

## Abbreviations

SLE: Systemic lupus erythematosus
HCQ: Hydroxychloroquine sulfate
NS: Normal saline
ELISA: Enzyme-linked immunosorbent assay
BUN: Blood urea nitrogen
ALB: Serum albumin
CCK-8: Cell Counting Kit-8
COVID-19: Coronavirus disease-2019
SARS-CoV-2: Severe acute respiratory syndrome coronavirus 2
